# Flaxseed Lignan Alleviates the Paracetamol-Induced Hepatotoxicity Associated with Regulation of Gut Microbiota and Serum Metabolome

**DOI:** 10.3390/nu16020295

**Published:** 2024-01-18

**Authors:** Yongyan Ren, Zhenxia Xu, Zhixian Qiao, Xu Wang, Chen Yang

**Affiliations:** 1 Key Laboratory of Oilseeds Processing of Ministry of Agriculture and Rural Affairs, Hubei Key Laboratory of Lipid Chemistry and Nutrition, Oil Crops Research Institute of the Chinese Academy of Agricultural Sciences, No. 2 Xudong 2nd Road, Wuhan 430062, China; 2 Institute of Hydrobiology, Chinese Academy of Sciences, No. 7 Donghu South Road, Wuhan 430060, China; 3 College of Animal Science and Technology, Huazhong Agricultural University, No. 1 Shizishan Street, Wuhan 430070, China

**Keywords:** paracetamol, liver injury, flaxseed lignan, gut microbiota, untargeted metabolome

## Abstract

This study examined the protective effect of flaxseed lignans on liver damage caused by an overdose of paracetamol (PAM). The findings demonstrated that administering 800 mg/kg/d flaxseed lignan prior to PAM significantly decreased the serum aspartate aminotransferase (AST), alanine aminotransferase (ALT), and total bilirubin (TBi) levels, while it increased liver superoxide dismutase (SOD) and glutathione (GSH) levels in mice. Flaxseed lignan renovated the gut microbiota dysbiosis induced by PAM by promoting the proliferation of sulfonolipid (SL) producing bacteria such as *Alistipes* and lignan-deglycosolating bacteria such as *Ruminococcus* while inhibiting the growth of opportunistic pathogen bacteria such as *Acinetobacter* and *Clostridium*. Furthermore, flaxseed lignan modulated the serum metabolomic profile after PAM administration, specifically in the taurine and hypotaurine metabolism, phenylalanine metabolism, and pyrimidine metabolism. The study identified eight potential biomarkers, including enterolactone, cervonyl carnitine, acutilobin, and PC (20:3(5Z, 8Z, 11Z)/20:0). Overall, the results suggest that flaxseed lignan can alleviate PAM-induced hepatotoxicity and may be beneficial in preventing drug-induced microbiome and metabolomic disorders.

## 1. Introduction

Paracetamol (PAM), also known as N-acetaminophen, is one of the most commonly used analgesic and antipyretic drugs in the world. As an available over-the-counter (OTC) drug in China, PAM is frequently used to mild pain and ease fever caused by virus infection, and many people even overtook it during the COVID-19 pandemic [1]. With the liver enzyme cytochrome P450 (Cyp2E1) and Cyp1A2PAM, PAM is converted into a highly reactive metabolite named acetyl para benzoquinoneimine (NAPQI) [2], which could be detoxified into non-toxic glutathione-NAPQI by conjugating with glutathione (GSH) [3]. However, in cases of paracetamol overdose, excess NAPQI depletes GSH stores, irreversibly binds to hepatocellular proteins, and causes oxidative damage by producing reactive oxygen species (ROS), resulting in hepatocellular apoptosis and necrosis [4,5]. Thus, PAM overdose or long-term consumption could induce oxidative stress-mediated hepatotoxicity which leads to severe liver injury or even death [6,7]. Therefore, developing effective antioxidants is a promising strategy to optimize the outcome of PAM toxicity.

Recent studies have shown that natural antioxidants such as polyphenols can be used to protect the liver from damage caused by PAM. One example is the use of dandelion polyphenol, which has been found to reduce oxidative stress and liver toxicity caused by PAM. This process is through the inhibition of the JNK signaling pathway and the activation of the Nrf-2/HO-1 pathway [8]. Flaxseed lignan is the dominant dietary polyphenols in flaxseed (*Linum usitatissimum*) [9]. Among various foods, flaxseed is the richest source of secoisolariciresinol diglucoside (SDG) (7 mg/g or 3.7 mg SECO 2/g), which was reported to display beneficial effects in reducing risk factors for obesity [10], hyperglycemia [11], and even cancers due to its superior antioxidant activity [12] and anti-inflammation activity [13].

Accumulative research suggested that the gut microbiota is a vital part of the mechanism of liver damage through the gut–liver axis [14]. Flaxseed lignan has poor bioavailability in the mammalian digestive tract but can be metabolized into the mammalian lignan-phytoestrogenic enteroligan enterodiol (END) and enterolactone (ENL) under the action of intestinal microbiota [15]. To a lesser extent, these compounds can effectively bind to glucuronic acid or sulfate free radicals in the intestinal epithelium and liver, and circulate via the enterohepatic circulation. Therefore, gut microbiota may be the most important factor to target for flaxseed lignan metabolism in order to implement or amplify its bioactivity.

The hepatoprotective effect of sesame ligan [16] and ligan from justicia simplex [17] against carbon tetrachloride-induced hepatotoxicity are well elucidated. As far as we know, we have little information about the extent to which flaxseed lignan can alleviate liver injury caused by PAM, and how it is related to changes in gut microbiota and metabolites. Given the anti-inflammatory, anticancer, and antitumor properties of flaxseed lignan, we propose that it can be used as a natural remedy to protect the liver. Therefore, in this research, we combined 16S rDNA microbiome analysis and serum metabolome techniques to investigate the protective effects of flaxseed lignan on PAM consumption-induced hepatoxicity and discuss the potential mechanism.

## 2. Materials and Methods

### 2.1. Chemicals and Kits

Flaxseed lignan (SDG 38%) was obtained from the Zhejiang Pan Weifeng Plant Material Factory (Jinhua, China). Paracetamol and silymarin were acquired from Macklin Biochemical Technology Co., Ltd. (Shanghai, China). The alanine aminotransferase (ALT), aspartate aminotransferase (AST) test kit, and the total bilirubin (TBi) detection kit in mouse serum were all purchased from Elabscience Biotechnology Co., Ltd. (Wuhan, China). The total protein content testing kit was purchased from Beyotime Biotech (Shanghai, China), while the malondialdehyde (MDA), glutathione (GSH), and superoxide dismutase (SOD) were detected by commercial kits from Nanjing Jiancheng Institute of Bioengineering (Nanjing, China). Other chemicals at analytical grade were purchased from Sinopharm Chemical Reagent Co., Ltd. (Shanghai, China).

### 2.2. Animal Experimental Design

The animal experiment involved 45 male C57BL/6 mice, 4–5 weeks of age and weighing 20-22 g, which were obtained from the Laboratory Animal Center of Sanxia University. Mice were kept in a specific pathogen free (SPF) room with 22 ± 2 °C, 50 ± 5% humidity, and a 12 h light/dark cycle. The mice were fed with the AIN93M standard diet (see Supplemental File Table S1) and water ad libitum. The experiments were approved by the Animal Ethics Committee of the Experimental Animal Center of Huazhong Agricultural University (HZAUMO-2021-0172).

After 7 days of acclimatization, the mice were randomly separated into five groups (*n* = 9) and treated as follows: (1) the low flaxseed lignan group and (4) the high flaxseed lignan groups received 400 mg/kg·BW/d and 800 mg/kg·BW/d flaxseed lignan gavage once a day for 14 days, respectively. (2) The SIM group received silymarin (50 mg/kg·BW) once a day for 14 days. (3) The PAM group received the same volume of saline solution by gavage once a day for 14 days. (4) On the 15th day, paracetamol was dissolved in saline (0.9% NaCl) and shaken well before use. The lSDG, hSDG, SIM, and PAM groups received single intra-gastric paracetamol (300 mg/kg·BW). (5) The NC group was given the same volume of saline by gavage throughout the 15-day experiment.

After 24 h, blood samples were collected from the orbital plexus. Serum was separated after centrifugation at 4000 rpm for 10 min and stored at −80 °C. According to AVMA guidelines for the euthanasia of animals (2020 Edition) [18], the mice were euthanized by spinal dislocation after carbon dioxide narcosis. The whole process is carried out by a skilled operator to minimize or eliminate the animals’ panic and pain. Liver tissue was removed immediately, and part of the liver tissue was placed in 4% paraformaldehyde. The left part was stored at −80 °C for further analysis. Fresh fecal samples were collected in sterilized Eppendorf tubes and immediately frozen at −80 °C.

### 2.3. Evaluation of Effect of Paracetamol

#### 2.3.1. Liver Damage

Serum enzyme levels of ALT, AST, and TBi were estimated using commercial kits (Nanjing Jiancheng Bio, Nanjing, China) according to the manufacturer’s protocols.

#### 2.3.2. Oxidative Stress

Fresh liver tissue was homogenized in 0.1 M PBS (1:9) and centrifuged at 12,000× *g* for 10 min. Levels of MDA, SOD, and GSH in the supernatant were determined using commercial kits (Nanjing Jiancheng Bio, Nanjing, China).

#### 2.3.3. Histopathological Studies

After 4% paraformaldehyde fixation, the liver tissue underwent gradient dehydration and was embedded with paraffin. The sample blocks were cut into 6 μm slices and stained with hematoxylin and eosin (H&E). The stained sections were observed and captured under the Eclipse Ts2R-FL microscope (Nikon, Tokyo, Japan).

#### 2.3.4. Bioinformatics Analysis of Gut Microbiota

The genomic DNA of the feces sample was isolated using a DNA extraction kit (CWBIO, Beijing, China), and the regions of 16Sr DNA V3-V4 regions were amplified with PCR primers 338F and 806R (338F, 5′ACTCCTACGGGAGGCAGCA-3′; 806R, 5′-GGACTACHVGGGTWTCTAAT-3′). The amplified products were sent to sequencing on the Illumina HiSeq platform (Illumina, San Diego, CA, USA) and the raw sequences were assigned to the operational taxonomic unit (OUT), in which the sequences had ≥97% similarity.

Alpha diversity was measured by the Chao1, Simpson, and Shannon indexes and was calculated through Quantitative Insights into Microbial Ecology (QIIME). Beta diversity was measured by principal coordinate analysis (PCoA) with unweighted UniFrac distances. We used Linear discriminant analysis (LDA) coupled with effect size was achieved using the LEfSe program (R3.6). The Spearman correlations between changed biochemical parameters, gut bacteria, and differential metabolites were analyzed with the R package (V2.15.3).

#### 2.3.5. Untargeted Serum Metabolomics Analysis

Serum sample and extract (methanol: acetonitrile = 1:1, *v*/*v*) were vortexed, followed by ultrasonication, centrifuged, and supernatants of the same amount from the whole samples were mixed into QC samples for further analysis. Chromatographic separation was carried out using the HPLC-Q-Orbitrap-MS HSS T3 column (100 mm × 2.1 mm, 1.8 µm; Thermo Fisher Scientific, Waltham, MA, USA). The mobile phase is 0.1% formic acid (mobile phase A) and 0.1% acetonitrile (mobile phase B). Then we started elution at a flow rate of 0.3 mL/min: 0–2 min, 95% A; 2–12 min, 5% A; 12–15 min, 5% A; 15–17,95% A. The ESI-MSn experiments were performed using positive and negative ion modes under 3.8 kV spray voltage in the positive mode and 3.2 kV spray voltage in the negative mode, the capillary temperature was 350 °C and the heater temperature was 300 °C, the airflow was 45 Arb, the auxiliary airflow was 10 Arb in positive mode and 5 Arb in the negative mode. The S-Lens F levels of positive and negative ion modes were 30% and 60%, respectively.

The LC/MS data were extracted and preprocessed by Compound Discoverer 2.1 software, and the mass, retention time, and peak intensity were exported. PCA and OPLS-DA analysis were performed on mouse serum samples using SMICA-P software (V16.0.2). We searched the HMDB, KEGG, and mzCloud databases to identify metabolites based on accurate mass and product ion spectra. MetaboAnalyst 4.0 was used for metabolic pathway analysis.

### 2.4. Statistical Analysis

All data were presented as the mean ± standard error (SE). Statistical analysis was performed using SPS 19.0. Differences between each group were analyzed by the one-way ANOVA (analysis of variance) test followed by Tukey’s post hoc test. It is considered a significant difference when the *p*-value was below 0.05.

## 3. Results

### 3.1. Flaxseed Lignan Alleviates Liver Injury Induced by PAM

**Figure 1:**
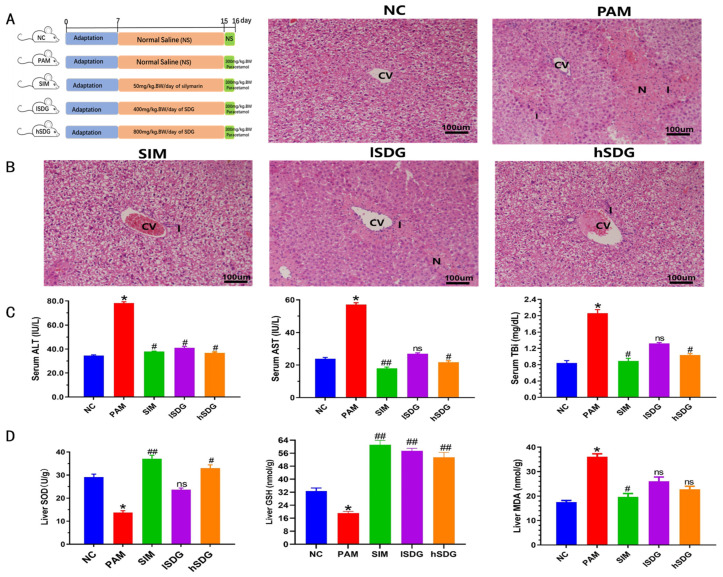
Effect of SIM and flaxseed lignan on liver function in PAM-induced hepatoxicity mice. (**A**) Diagram of research experimental design. (**B**) Photomicrograph of hepatic tissue (200×, scale bar: 100 μm). (**C**) Serum levels of ALT, AST, and Tbi. (**D**) MDA, SOD, and GSH levels in the liver tissue. CV: central vein; N: necrosis; I: inflammation. * means *p* < 0.05 when compared with NC group; # and ## means *p* < 0.05 and *p* < 0.01 when compared with the PAM group, respectively; ns means *p* > 0.05 when compared with the PAM group.

The experimental design for the animal study is depicted in Figure 1A. Histopathological analysis of the liver tissue revealed that the normal control group (NC group) exhibited normal hepatic cells with intact cell structure (Figure 1B). In contrast, the group exposed to PAM without any pre-treatment (PAM group) showed extensive necrosis in the perivenular and midzonal regions, as well as infiltration of lymphocytes. The group pre-treated with 400 mg/kg flaxseed lignan (lSDG group) displayed mild inflammation and necrotic regions. The 800 mg/kg flaxseed lignan-treated group (hSDG group) resulted in mild inflammation but no necrosis, as well as in the group pre-treated with silymarin (SIM group).

To investigate the impact of flaxseed lignan on PAM-induced liver injury, biochemical indexes of serum and liver tissue were evaluated (Figure 1C,D). PAM administration led to a significant increase in the levels of ALT, AST, and TBi in serum when compared to the NC group (*p* < 0.05; *p* < 0.05; *p* < 0.05). However, pretreatment with 50 mg/kg SIM gradually decreased the activities of these three enzymes (*p* < 0.05; *p* < 0.01; *p* < 0.05). Similarly, the hSDG group exhibited decreased these enzyme activities (*p* < 0.05; *p* < 0.05; *p* < 0.05). On the other hand, the lSDG group only reduced the activity of ALT and did not affect AST or TBi levels compared to the PAM group.

The activities of SOD and GSH from liver homogenate in the PAM group were significantly lower than those in the NC group (*p* < 0.05; *p* < 0.05). However, both SIM and hSDG groups showed enhanced SOD (*p* < 0.01; *p* < 0.05) and GSH (*p* < 0.01; *p* < 0.05) activities compared to the PAM group. The lSDG group also exhibited a significant increase in GSH levels (*p* < 0.01). The level of MDA, a marker of oxidative stress, was significantly elevated in the PAM-induced mice compared to the NC mice (*p* < 0.05), but only the SIM group reduced the MDA levels (*p* < 0.05).

### 3.2. Flaxseed Lignan Regulates Gut Microbiota Structure Induced by PAM

**Figure 2:**
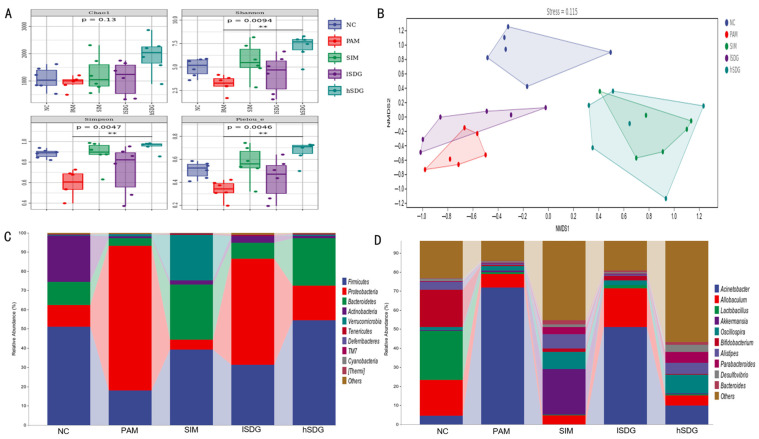
Effects of SIM and flaxseed lignan on microbial diversity and structure in the fecal samples. (**A**) α-diversity indexes are presented by box plot. (**B**) Non-metric multidimensional scaling (NMDS) result based on unweighted UniFrac distance matrices. (**C**) The composition of the top 10 gut microbiota at the phylum level. (**D**) The composition of the top 10 gut microbiota at the genus level. ** means *p* < 0.001 when compared with the PAM group.

16S rDNA gene sequencing was conducted on fecal microbiota to investigate the impact of pretreatment with flaxseed lignan on the intestinal microbiota. The diversity of the intestinal microbial community was evaluated using alpha-diversity and beta-diversity measures. As shown in Figure 2A, the Simpson, Shannon, and Pielou’s evenness indexes of the PAM group were significantly lower than those of the NC group. However, administration of SIM or a higher dosage of flaxseed lignan (hSDG group) noticeably increased these indexes. No significant differences were observed in the Chao1 index among the five groups. These findings suggest that pretreatment with a high dosage of flaxseed lignan enhances the evenness and richness diversity of the microbiota community.

A non-metric multidimensional scale (NMDS) map based on the weighted UniFrac phylogenetic distance matrix was used to compare the intestinal microbiota characteristics in the five groups. As depicted in Figure 2B, the intestinal microbial community of the PAM group exhibited distinct characteristics compared to the NC group, with notable similarities observed between the PAM and flaxseed lignan groups. Furthermore, the gut microbiota pattern of the hSDG group was more closely aligned with the SIM group than the lSDG group.

The figure presented in Figure 2C displays the top 10 phylum taxa in the gut microbiota among five groups. The most abundant phyla of all groups were Firmicutes, Bacteroidetes, Proteobacteria, and Actinobacteria. Compared to the NC group, the PAM group showed a higher abundance of Proteobacteria but a lower abundance of Firmicutes, Bacteroidetes, and Actinobacteria. With the exception of Actinobacteria, pre-administration of flaxseed lignan and silymarin reversed this trend. At the genus level, as shown in Figure 2D, the PAM group exhibited a significantly increased abundance of *Acinetobacter*, which corresponds to the high phylum of Proteobacteria. It also decreased the abundance of *Allobaculum*, *Akkermansia*, *Allistipes*, *Lactobacillus*, and *Bifidobacterium* compared to the NC group. Both the SIM and SDG groups restored the abundance of *Allistipes* and suppressed the abundance of *Acinetobacter*. Additionally, while the SIM pretreatment restored the decreased *Akkermansia*, flaxseed lignan repaired the decreased *Allobaculum* compared to the PAM group. Notably, the flaxseed lignan group was unable to regulate those genera in PAM-induced mice, except for *Allobaculum*.

**Figure 3:**
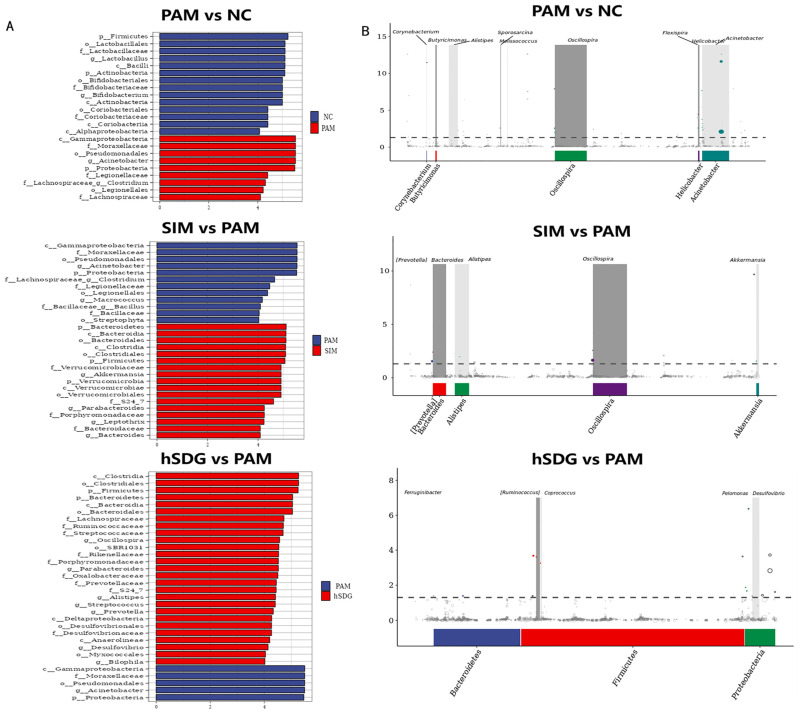
Analysis of significantly regulated intestinal microbiota by Lefse (**A**) and Metastats (**B**) between the two groups. Data (*n* = 9) are expressed as mean ± SEM. Only taxa meeting an LDA significant threshold > 4 are shown.

Additionally, the study employed linear discriminant analysis (LDA) effect size (LEfSe) to identify potential biomarkers that could indicate liver injury associated with changes in gut microbiota (Figure 3A). The presence of *Acinetobacter* and *Clostridium* was significantly higher in the PAM group compared to the NC group, which had higher levels of *Lactobacillus* and *Bifidobacterium*. On the other hand, the SIM group showed enrichment of *Akkermansia*, *leptothrix*, and *Bacteroides*. In the lSDG group, *Allobaculum* and *Bifidobacterium* were found to be enriched, while *Oscilospira*, *Parabacteroides*, *Prevotella*, and *Bilophila* were significantly enriched in the hSDG group. Furthermore, Metastats analysis was conducted to assess the differences between the two groups at various taxonomic levels, and the results were consistent with those obtained from the LEfSe analysis (Figure 3B).

### 3.3. Flaxseed Lignan Alters Serum Metabolome Composition Induced by PAM

**Figure 4:**
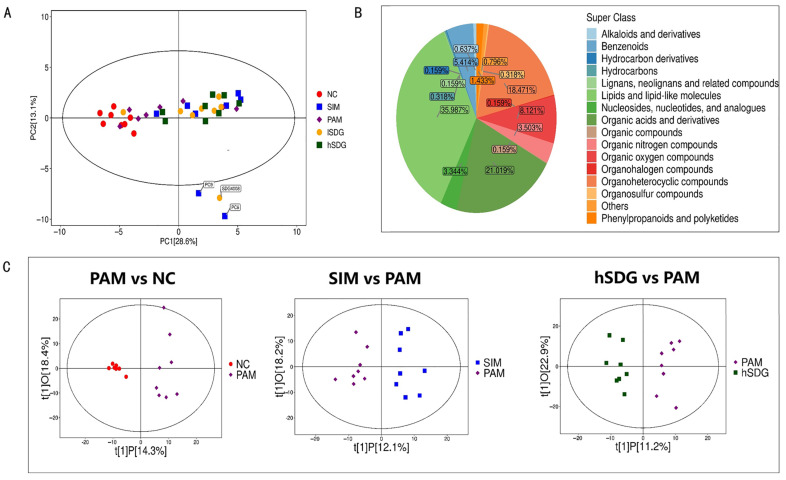
Multivariate statistical analysis of untargeted metabolomics data in the serum samples. (**A**) PCA score plot based on the combined positive and negative model data. (**B**) Superclass distribution of detected metabolites in all samples. (**C**) OPLS-DA analysis of three comparison groups: PAM vs. NC (**left**), SIM vs. PAM (**middle**), and flaxseed lignan vs. PAM (**right**).

The metabolic profiles of serum samples were analyzed using HPLC-MS to determine the presence of positive and negative ions. The results are shown in Figure 4A, indicating a distinct separation of serum metabolites in the samples from the NC, PAM, SIM, and two SDG groups. Figure 4B summarizes the distribution of metabolites detected in all samples, with ligan, neoligan, and related compounds accounting for 35.99% of the total metabolites, followed by organic acids and derivatives at 21.1%, and organoheterocyclic compounds at 18.47%. These results were further analyzed using OPLS-DA plots to establish pairwise group comparisons. The separation between each two-pair group is clearly depicted in Figure 4C, indicating significant changes in the metabolic phenotype in mice. The OPLS-DA model validation yielded interpretation ability parameters (R2Y) ranging from 0.923 to 0.949 and prediction ability parameters (Q2) ranging from 0.525 to 0.603, indicating the validity of the models.

**Figure 5:**
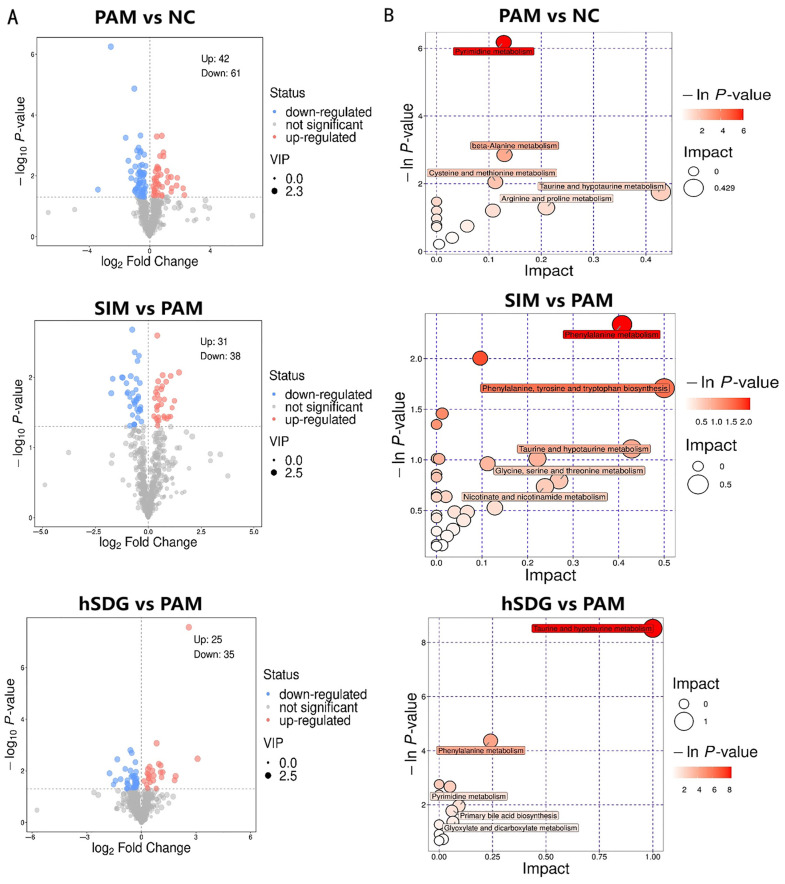
Differently expressed serum metabolites and pathways for SIM and flaxseed lignan pretreatment mice (positive ion combined with negative ion). (**A**) Volcano plots for the model-separated metabolites following the conditions of VIP > 1 and *p* (corr) < 0.01 with 95% jackknifed confidence intervals. PAM vs. NC (**upper**), SIM vs. PAM (**middle**), and flaxseed lignan vs. PAM (**bottom**). (**B**) Bubble plots for the KEGG pathway enrichment of the changed metabolites between each compared group. PAM vs. NC (**upper**), SIM vs. PAM (**middle**), and flaxseed lignan vs. PAM (**bottom**).

Candidate biomarkers were selected based on VIP > 1 and *p* < 0.05 criteria and are displayed in volcano plots in Figure 5A. A total of 103 significantly changed metabolites were identified between the PAM and NC groups, of which 42 were upregulated and 61 were downregulated. In comparison to the PAM group, there were 31 significantly upregulated metabolites and 38 significantly downregulated metabolites identified in the SIM group. Similarly, the hSDG group exhibited 25 significantly upregulated metabolites and 35 significantly downregulated metabolites when compared to the PAM group. These metabolites were characterized as lipids and lipid-like molecules, organic acids and derivatives, and organic oxygen compounds (see Supplemental File Figure S1).

In this study, we focused on the metabolites which were significantly regulated both in SDG and PAM groups. The results revealed 16 metabolites that were significantly modulated, indicating their potential as biomarkers for flaxseed lignan pre-administration in mice with liver damage. As shown in Table 1, these metabolites primarily belonged to the categories of lipids and lipid-like molecules, organic oxygen compounds, organic acids and derivatives, phenylpropanoids and polyketides, and indole-3-propionic acid.

To further understand the effects of SIM or flaxseed lignan on metabolic pathways in mice, all the differential metabolites were subjected to KEGG enrichment analysis. As depicted in Figure 5B, SIM treatment significantly altered six metabolic pathways, including taurine and hypotaurine metabolisms, arginine and proline metabolism, alanine, aspartate and glutamate metabolism, and pyrimidine metabolism. On the other hand, the hSDG group affected the taurine and hypotaurine metabolism, phenylalanine metabolism, pyrimidine metabolism, and nine other pathways when compared to PAM group. The enriched KEGG pathways and the representative changed metabolites are listed in Table 2.

### 3.4. Regulation of Gut Microbiota Is Associated with Metabolome Alteration

**Figure 6:**
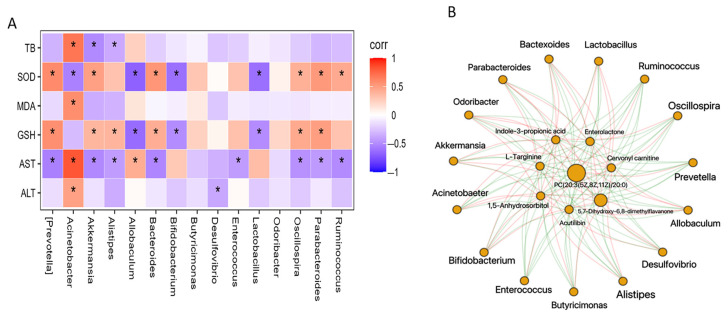
Correlation among regulated gut microbiota, biochemical parameters, and changed metabolites. (**A**) Heatmap of Spearman’s correlation between changed bacterial taxa and biochemical parameters. (**B**) Pie network of Spearman’s correlation between changed bacterial taxa and regulated metabolites. Significant differences are indicated as * (*p* < 0.05). Red lines indicate significantly positive correlation, green lines indicate significantly negative correlation.

Spearman correlation analysis was conducted to assess the potential connections between significantly altered bacteria in the gut microbiome and the parameters related to transaminases and oxidative stress (Figure 6A). The findings revealed that 15 bacterial genera were either positively or negatively associated with at least one biochemical marker. *Acinetobacter*, for instance, showed positive associations with ALT, AST, MDA, and TBi, but a negative association with SOD. Among all the genera, SOD, GSH, and AST exhibited the highest correlations. Specifically, GSH and SOD were positively correlated with *Akkermansia*, *Bacteroides*, *Oscillospira*, and *Parbacteroides*, but negatively associated with *Allobaculum*, *Bifidobacterium*, and *Lactobacillus*. Conversely, GSH displayed an opposite correlation with these genera. TB showed a positive correlation with *Acinetobacter*, but a negative correlation with *Akkermansia* and *Alistipes*.

Furthermore, Spearman correlation analysis was performed to examine the relationship between altered metabolites and the changed biochemical parameters (Supplemental File Figure S2). Eight potential biomarkers were identified to have a strong association with transaminases and oxidative stress index. Subsequently, the information regarding the selected correlated metabolites and the 15 altered genera was utilized to construct the pie network. As depicted in Figure 6B, acutilobin, L-targinine, and enterolactone were found to have a negative correlation with *Acinetobacter*, *Allobaculum*, *Lactobacillus*, and *Bifidobacterium*, while displaying a positive correlation with the other 11 genera. On the other hand, indole-3-propionic acid exhibited an opposite trend. Additionally, 5, 7-Dihydroxy-6, 8-dimethyl-flavanone, PC (20:3 (5Z, 8Z, 11Z)/20:0), and 1,5-Anhydrosorbitol were positively associated with all the selected genera.

## 4. Discussion

Recently, medicinal plants and plant-derived compounds have attracted worldwide attention in the treatment of various liver diseases [19]. Flaxseed lignan, termed lignan macromolecule, is mainly composed of SDG structures held together by four hydroxy-methylglutaric acid (HMGA) residues [20]. It can be converted into active enteroligan, such as END and ENL by flaxseed lignan-deglycosolating bacteria, which are characterized by various biological activities, including activation of tissue-specific estrogen receptors and anticancer and anti-inflammatory effects [21].

Previous studies suggested the safety of flaxseed lignans and confirmed their metabolites may possess anticancer properties. Among them, the SDG is the main form of flaxseed lignan which showed no cytotoxicity even at concentrations exceeding 1000 µM in either cancerous or non-cancerous cell lines [22]. An in vivo study of continuous intake of lignan 40 mg/kg for two months showed no negative effects on the hematological system in both normal and hypercholesterolemic rabbits [23]. Human participants were also shown to be safe and well tolerated by a complex containing 34% to 38% SDG, which was administered at a dosage of 600 mg/day for 6 months [24].

The safety of flaxseed lignan in this study was also confirmed in our preliminary experiment, in which no obvious evidence of damage in the liver or intestinal canal was found when flaxseed lignan was administrated at the dosage of 800 mg/kg·BW for 14 days. Thus, in this study, the researchers investigated the hepatoprotective activity of flaxseed lignan on liver injury induced by paracetamol, as well as its effects on gut microbiota and serum metabolome in mice.

Paracetamol causes elevated serum AST and ALT levels in mice, which are commonly used as indicators of liver injury [25]. The increase in AST is usually accompanied by a rising level of ALT, which plays a crucial role in the conversion of amino acids to ketoacids [26]. The administration of paracetamol led to an increase in the levels of serum AST and ALT in mice, which are commonly used as indicators of liver injury [27]. Disruption of the transport mechanism in hepatocytes can result in the leakage of the plasma membrane, leading to elevated levels of serum enzymes [28]. These findings were also observed in mice that were not pre-treated, as paracetamol consumption alone caused an increase in total bilirubin levels in the serum. However, oral administration of silymarin at a dose of 50 mg/kg body weight protected against paracetamol-induced liver damage by reducing the levels of AST, ALT, and total bilirubin, as previously reported. Interestingly, mice pre-treated with a higher dose of flaxseed lignan at 800 mg/kg also showed significant reversal of these changes, demonstrating a comparable activity to the control drug silymarin at 50 mg/kg.

Oxidative stress is closely associated with harmful paracetamol-induced liver damage, specifically involving the cytochrome P450 (CYP) system [29]. The liver antioxidant enzymes SOD and GSH play an important role in balancing redox status. However, endogenous supplementation of GSH alone is insufficient to fully alleviate the hepatotoxicity caused by paracetamol [30]. In this study, serum GSH and SOD were higher in silymarin-treated mice, which is in agreement with previous studies demonstrating improved antioxidant status [31]. In our study, we found that pretreatment with flaxseed lignan significantly increased GSH and SOD levels in mice with paracetamol-induced liver injury, similar to the effects observed in the silymarin group. This suggests that flaxseed lignan supplementation positively affects antioxidant enzymes in mice with paracetamol-induced liver injury, potentially preventing hepatonecrotic damage caused by excessive paracetamol dosage.

Dietary lignans such as flaxseed lignan are known poorly absorbed in the small intestine and reach the colon undigested [32]. Ellen et al. investigated the fate of the flaxseed lignan macromolecule in the gastrointestinal lumen and found the main chains of lignan macromolecules depolymerize to different degrees, and the phenolate glycosides attached to SDG glycosides are released during gastric digestion. Moreover, no more changes and no more SECO were detected after the stomach and small intestinal digestion [33]. Another Study determined the bioaccessibility values of SECO, ED, and EL in whole flaxseed were 0.75%, 1.56%, and 1.23%, respectively [34]. These results suggested flaxseed lignans exert poor bioaccessibility to small intestinal cells, and a large portion of them are converted into END and ENL by the intestinal microbiota [35]. These enterolignans are much more bioavailable than their lignan precursors (SDG or SECO) [36]. Due to its low bioavailability, its anticancer biological activities depend on the bacterial transformation efficiency occurring in the colon [37].

It was also reported that the molecular structure is a critical factor for the antioxidative activity of flaxseed lignans [38]. Both flaxseed lignan precursors and their metabolites were reported to have antioxidative activities. Due to the presence of the 4-hydroxy-3-methoxyphenylpropanol structure, SDG and SECO have superiority in antioxidative activity compared to their metabolites END and ENL, as SDG and SECO effectively scavenge DPPH free radicals at concentrations of 25–200 μM [39]. Combined with the fate of flaxseed lignan in the mammals’ digestive tract mentioned above, a very small amount of SDG and SECO are absorbed by the small intestine, and most of the flaxseed lignans are converted into END and ENL and absorbed through enterohepatic circulation. Therefore, we speculated that the improvement of oxidative stress in the liver is possibly due to the ability of both these compounds to scavenge free radicals, but which substance has a larger effect needs to be further investigated.

Furthermore, recent research has focused on understanding the relationship between gut microbiota and low-grade inflammation, which can lead to organ injury, including in the colon [40] and liver. Pro-inflammatory cytokines, such as tumor necrosis factor α (TNF-α), interleukin 1β (IL-1β), and interleukin 6 (IL-6), were commonly used as biomarkers to reflect the inflammatory state in serum or tissue in animal experiments. Mohamed et al. reported that a single intragastric dose of paracetamol (500 mg/kg·BW) increased the gene expression of IL-1β, IL-8, and TNF-α in the liver [41]. Maria et al. also reported that orally administrated 2 g/kg.BW paracetamol in Wistar rats determined an increase in the serum levels of TNF-α and IL-1β compared with the control group [42]. However, we failed to detect any significant changes among serum IL-1β, IL-6, or TNF-α in the preliminary experiment. This might be due to the differences between drug dosage and test animals.

In our study, we observed that supplementation of flaxseed lignan for 14 days, along with inducing hepatic injury in mice treated with paracetamol, resulted in an altered composition of intestinal bacteria. This composition was distinct from the normal control group and the group treated with silymarin. Similar to silymarin, flaxseed lignan pretreatment also increased the richness and evenness of microbial diversity. Plant lignan has the ability to modulate the composition of intestinal microbes, and in turn, these microbes break down lignan to release bioactive metabolites [43]. As expected, the unabsorbed lignan was utilized as substrate by enterolactone-producing bacterial species, thus improving the gut microbiota diversity. These results were also observed in normal-fed mice models [44,45] and an in vitro pilot study [46]. Our results suggest that flaxseed lignan can modulate the composition of gut microbiota in the presence of paracetamol-induced liver injury.

A remarkable increase in the abundance of the *Acinetobacter* and *Clostridium* genus was detected after PAM administration. The genus *Acinetobacter* is a highly diverse group, which comprises species with ecological significance and opportunistic pathogens [47]. Several *Acinetobacter* species are confirmed as opportunistic pathogens causing various human infections [48]. Coincidentally, previous research also demonstrated that *Clostridium* species (particularly *Clostridium difficile*, *Clostridium botulinum*, *Clostridium tetani*, and *Clostridium perfringens*) are related to a range of human and animal diseases [49]. Our results showed that PAM promoted the growth of *Acinetobacter* and *Clostridium* in the gut, which may highly increase the risk of bacterial infection in untreated mice, whereas the pretreatment with silymarin and flaxseed lignan inhibited the proliferation of these two genera. Furthermore, beneficial bacteria such as *Lactobacillus* and *Bifidobacterium* were decreased after PAM overdose, and so did the bacterial sulfonolipids (SLs) producing genus *Alistipes* [50]. Pretreatment with silymarin and flaxseed lignan restored the *Alistipes* and increased the abundance of *Ruminococcus.* Several species of the *Ruminococcus* genus were reported to have the ability to transform dietary flaxseed lignan into animal lignan [22]. Moreover, only the supplementation of SIM promoted the growth of the promising probiotic *Akkermansia*, which is known to play a vital role in improving the host’s immune responses and metabolic functions [51]. Our findings demonstrated that similar to silymarin, the alleviation effect of flaxseed lignan on liver injury was also associated with the modulation of intestinal microbiota, although the specific bacteria the taxa targeted differed. These results were further supported by lefse_LDA analysis.

Various natural plant polyphenols with antioxidant properties are commonly used as food and medicine to target various diseases, particularly liver diseases [52,53]. While several studies have provided multifaceted mechanistic insights into the hepatoprotective effect of lignan derived from different plants [54,55], the relationship between gut microbiota, metabolic events, and the hepatoprotective effect of flaxseed lignan on the progression of PAM-induced liver injury has not been extensively explored.

This study aimed to analyze the serum metabolome to understand the specific metabolites that contribute to the reduction of liver injury caused by flaxseed lignan. This is the first study to investigate the serum metabolic profile of flaxseed lignan administration in PAM-induced liver damage mice. The distribution pattern of samples in the OPLS-DA score plot and volcanic scatter plot indicated a significant change in metabolic profiling due to PAM-induced liver injury.

Furthermore, the pattern of modification was different from SIM, indicating a unique mechanism that targets different pathways, as seen in previous studies. The study observed that compared to the negative control group, the pathways involved in “Taurine and Hypotaurine metabolism”, “Beta-alanine metabolism”, and “Arginine and Proline metabolism” were altered in response to PAM-induced hepatotoxicity. However, when the mice were pre-treated with flaxseed lignan, the pathways of “Taurine and Hypotaurine metabolism” and “Phenylalanine metabolism” were more prominent. It is known that liver diseases or acute liver injury can disrupt the metabolism of amino acids such as arginine, taurine, and glutamine, leading to lipid metabolism disorders [56]. Among them, taurine and L-targinine were well known as they exert therapeutic effects via anti-oxidation and anti-inflammation properties [57]. Moreover, gut-microbial organisms and their metabolites are involved in regulating oxidative stress and inflammation, which play vital roles in drug-induced hepatotoxicity [58]. The analysis of enriched metabolic pathways suggests that “Taurine and Hypotaurine metabolism” is the most affected pathway induced by PAM, and flaxseed lignan supplementation could alleviate this disturbance. In this study, 3-sulfinoalanine, taurine, and hypotaurine were identified as metabolic biomarkers for the PAM group, indicating that taurine metabolism disorder may be involved in the pathophysiology of PAM-induced hepatotoxicity and could be a targeted metabolic pathway for plant lignan intervention therapy.

The Phenylalanine metabolic pathway is a pro-inflammatory pathway commonly found in animals, plants, and microorganisms. The study found that treatment with flaxseed lignan decreased the levels of PC (20:3(5Z,8Z,11Z)/20:0) and 1,5-Anhydrosorbitol, which are involved in the phenylalanine pathway. 1,5-Anhydroglucitol is a biomarker for short-term glycemic control and has been verified as a sensitive and specific biomarker for diabetes [59]. Pretreatment with flaxseed lignan decreased the levels of PC (20:3(5Z,8Z,11Z)/20:0) and 1,5-Anhydrosorbitol, suggesting that flaxseed lignan may improve the pathological state of fatty liver by regulating lipid and organic oxygen compound metabolism.

Additionally, the consumption of flaxseed lignan increased the levels of cervonyl carnitine and flaxseed-derived polyphenol-enterolactone (ENL), which have been reported to affect membrane fatty acid transporters and regulate lipid metabolism for the treatment of non-alcoholic fatty liver disease (NAFLD) [60]. ENL undergoes extensive glucuronidation in the liver to form O-glucuronides, which decreases its potential toxicity [61]. It has been found to inhibit ß-oxidation and intracellular lipid metabolism, contributing to the progression of NAFLD [62]. Reduced fecal microbial metabolite enterolactone is considered a novel biomarker associated with inflammatory and oxidative stress in alcoholic liver disease [63]. Therefore, the increased content of ENL in the flaxseed lignan group suggests that flaxseed lignan attenuates PAM-induced hepatotoxicity by improving oxidative state and fatty acid metabolism. In summary, the mechanism by which flaxseed lignan reduces liver injury induced by PAM may involve the regulation of metabolic disorders.

## 5. Conclusions

To summarize, our research indicated that flaxseed lignan exhibits a protective effect against liver damage caused by PAM in mice. This protection is likely due to the restoration of aminotransferase levels (AST and ALT) and reduction of oxidative stress (SOD and GSH), as well as the preservation of hepatic cells. Additionally, flaxseed lignan influences the composition of gut microbiota by promoting the production of sulfonolipids (SLs) by the genus *Alistipes* and bacteria that break down flaxseed lignan, such as *Ruminococcus*. It also inhibits the growth of opportunistic pathogenic bacteria such as *Acinetobacter* and *Clostridium*. These changes in gut microbiota may be related to alterations in metabolites, including taurine and hypothalamine metabolism, as well as the phenylalanine pathway metabolism. This correlation between microbiota and metabolites offers a new perspective for investigating the preventive effects of flaxseed lignan. Overall, our findings demonstrate that flaxseed lignan has significant potential in mitigating PAM-induced liver injury in mice.

## Figures and Tables

**Table 1:** The potential biomarkers identified in the SDG pretreatment mice.

Metabolite	m/z	RT (min)	Classification	lSDG vs. PAM			hSDG vs. PAM		
				VIP	FC	Trend	VIP	FC	Trend
Indole-3-propionic acid	188.07	93.08	Organoheterocyclic compounds	1.81	0.407	↓	1.569	0.26	↓
1,5-Anhydrosorbitol	163.06	311.1	Organic oxygen compounds	2.55	0.464	↓	1.734	0.582	↓
PC(20:3(5Z,8Z,11Z)/20:0)	840.64	155.7	Lipids and lipid-like molecules	1.8	0.484	↓	1.889	0.454	↓
Armillaramide	556.53	87.9	Lipids and lipid-like molecules	2.17	0.616	↓	1.76	0.681	↓
Glutamyllysine	276.16	514.5	Organic acids and derivatives	1.9	0.709	↓	1.957	0.773	↓
Threonic acid	135.03	324.8	Organic oxygen compounds	2.35	0.732	↓	2.163	0.753	↓
D-2,3-Dihydroxypropanoic acid	105.02	196.7	Organic oxygen compounds	2.41	0.74	↓	1.927	0.71	↓
N-methyl-L-glutamic acid	162.08	345.7	Organic acids and derivatives	2.38	0.746	↓	1.688	0.784	↓
Imidazoleacetic acid riboside	259.09	367.1	Nucleosides, nucleotides, and analogs	1.69	0.75	↓	2.239	0.659	↓
Santene	123.12	33.86	Hydrocarbons	2.57	1.209	↑	2.157	1.287	↑
Ethylbenzene	107.09	33.85	Benzenoids	1.65	1.257	↑	2.183	1.366	↑
Acutilobin	329.14	147.5	Phenylpropanoids and polyketides	2.11	1.521	↑	2.164	2.085	↑
L-Targinine	189.13	543.5	Organic acids and derivatives	2.05	1.625	↑	2.385	1.79	↑
Cervonyl carnitine	472.34	196.3	Lipids and lipid-like molecules	1.79	2.984	↑	1.856	2.255	↑
5,7-Dihydroxy-6,8-dimethylflavanone	285.11	25.78	Phenylpropanoids and polyketides	2.21	3.862	↑	2.515	6.102	↑
Enterolactone	299.13	205.6	Ligan, neoligan, and related compounds	2.03	5.274	↑	2.394	8.512	↑

RT: Retention Time; VIP: Variable Important in Projection; FC: Fold Change; ↓: down-regulated; ↑: up-regulated.

**Table 2:** KEGG pathway enrichment of the changed metabolites for PAM vs. NC, SIM vs. PAM, and hSDG vs. PAM.

	KEGG Pathway	Impact	Changed Metabolite	KEGG No.	VIP	*p*-Value	Up/Down
NC-PAM	Taurine and hypotaurine metabolism	0.429	Taurine	cpd:C00245	1.45	0.04469	down
	Arginine and proline metabolism	0.222	Ornithine	cpd:C00077	1.90	0.00633	up
			L-Aspartic acid	cpd:C00049	1.48	0.04099	up
			L-Arginine	cpd:C00062	1.85	0.00371	down
			Creatine	cpd:C00300	1.54	0.02381	up
	Alanine, aspartate, and glutamate metabolism	0.193	L-Aspartic acid	cpd:C00049	1.48	0.04099	up
	Pyrimidine metabolism	0.139	Uridine	cpd:C00299	1.55	0.01971	down
			Cytidine	cpd:C00475	1.68	0.01047	down
			Thymidine	cpd:C00214.	1.40	0.04733	up
			Dihydrothymine	cpd:C00906.	1.69	0.03190	up
			Uracil	cpd:C00106	1.63	0.01816	down
	Beta-Alanine metabolism	0.130	L-Aspartic acid	cpd:C00049	1.48	0.04099	up
			3-Aminopropionaldehyde	cpd:C05665	1.48	0.03089	up
			Uracil	cpd:C00106	1.63	0.01816	down
hSDG-PAM	Taurine and hypotaurine metabolism	1.00	3-Sulfinoalanine	cpd:C00606	2.18	0.01294	down
			Taurine	cpd:C00245	1.93	0.02580	down
			Hypotaurine	cpd:C00519	2.13	0.01344	down
	Phenylalanine metabolism	0.241	Phenylpyruvic acid	cpd:C00166	1.73	0.01437	up
			Phenylacetylglycine	cpd:C05598	1.70	0.04416	down
	Pyrimidine metabolism	0.093	Cytidine	cpd:C00475	1.78	0.03234	down
			dUMP	cpd:C00365	1.84	0.02347	down
	Primary bile acid biosynthesis	0.060	Taurine	cpd:C00245	1.93	0.02580	down
			Taurochenodesoxycholic acid	cpd:C05465	1.62	0.03692	up
	Cysteine and methionine metabolism	0.051	5’-Methylthioadenosine	cpd:C00170	1.65	0.03000	down
			3-Sulfinoalanine	cpd:C00606	2.18	0.01294	down
SIM-PAM	Glycine, serine and threonine metabolism	0.269	Glycine	cpd:C00037	1.51	0.03110	down
	Nicotinate and nicotinamide metabolism	0.238	Niacinamide	cpd:C00153	1.97	0.01476	up
	Primary bile acid biosynthesis	0.030	Glycine	cpd:C00037	1.51	0.03110	down
	Glycerophospholipid metabolism	0.023	Glycerophosphocholine	cpd:C00670	1.29	0.04526	up
	Pantothenate and CoA biosynthesis	0.020	Pantothenic	cpd:C00864	2.25	0.03816	down

## Data Availability

The sequencing data have been deposited into a publicly accessible NCBI repository (accession: PRJNA932542).

## Supplementary Materials

The following supporting information can be downloaded at: https://www.mdpi.com/article/10.3390/nu16020295/s1, Figure S1: Superclass distribution of significantly changed metabolites in each pair group; Figure S2: Heatmap of Spearman’s correlation between changed metabolites and biochemical parameters; Table S1: The composition of standard diet AIN93M.

## References

[1] Liao J.Q., Lu Q.X., Li Z.Q., Li J.T., Zhao Q., Li J. (2023). Acetaminophen-induced liver injury: Molecular mechanism and treatments from natural products. Front. Pharmacol..

[2] Kaushal R., Dave K.R., Katyare S.S. (1999). Paracetamol hepatotoxicity and microsomal function. Env. Toxicol. Phar..

[3] Locigno R., Pincemail J., Henno A., Treusch G., Castronovo V. (2002). S-acetyl-glutathione selectively induces apoptosis in human lymphoma cells through a GSH-independent mechanism. Int. J. Oncol..

[4] Saito C., Lemasters J.J., Jaeschke H. (2010). c-Jun N-terminal kinase modulates oxidant stress and peroxynitrite formation independent of inducible nitric oxide synthase in acetaminophen hepatotoxicity. Toxicol. Appl. Pharm..

[5] Prozialeck W.C. (2009). Toxicology and Applied Pharmacology. Foreword. Toxicol. Appl. Pharmacol..

[6] Jaeschke H., McGill M.R., Williams C.D., Ramachandran A. (2011). Current issues with acetaminophen hepatotoxicity-A clinically relevant model to test the efficacy of natural products. Life Sci..

[7] Toklu H.Z., Sehirli A.O., Velioglu-Ogunc A., Cetinel S., Sener G. (2006). Acetaminophen-induced toxicity is prevented by beta-D-glucan treatment in mice. Eur. J. Pharmacol..

[8] Ren Y.S., Zheng Y., Duan H., Lei L., Deng X., Liu X.Q., Mei Z.N., Deng X.K. (2020). Dandelion polyphenols protect against acetaminophen-induced hepatotoxicity in mice via activation of the Nrf-2/HO-1 pathway and inhibition of the JNK signaling pathway. Chin. J. Nat. Med..

[9] Mueed A., Shibli S., Jahangir M., Jabbar S., Deng Z.Y. (2022). A comprehensive review of flaxseed (*Linum usitatissimum* L.): Health-affecting compounds, mechanism of toxicity, detoxification, anticancer and potential risk. Crit. Rev. Food Sci..

[10] Wu Z.Y., Wu B.F., Lv X., Xie Y., Xu S.L., Ma C.C., Xu J.Q., Tu X.H., Wei F., Chen H. (2021). Serumal Lipidomics Reveals the Anti-inflammatory Effect of Flax Ligan and Sinapic Acid in High-Fat-Diet-Fed Mice. J. Agric. Food Chem..

[11] Tse T.J., Guo Y.J., Shim Y.Y., Purdy S.K., Kim J.H., Cho J.Y., Alcorn J., Reaney M.J.T. (2022). Availability of bioactive flax lignan from foods and supplements. Crit. Rev. Food Sci..

[12] Newairy A.S.A., Abdou H.M. (2009). Protective role of flax ligan against lead acetate induced oxidative damage and hyperlipidemia in rats. Food Chem. Toxicol..

[13] Kim K.H., Moon E., Kim S.Y., Choi S.U., Lee K.R. (2012). Lignan constituents of Tilia amurensis and their biological evaluation on antitumor and anti-inflammatory activities. Food Chem. Toxicol..

[14] Coskun A.B., Celep A.G.S. (2022). Therapeutic modulation methods of gut microbiota and gut-liver axis. Crit. Rev. Food Sci..

[15] Mueed A., Ibrahim M., Shibli S., Madjirebaye P., Deng Z.Y., Jahangir M. (2022). The fate of flaxseed-ligan after oral administration: A comprehensive review on its bioavailability, pharmacokinetics, and food design strategies for optimal application. Crit. Rev. Food Sci..

[16] Chen W.L., Lu H.C., Huang H.Y., Hwang G.Y., Tzen J.T.C. (2010). Sesame Ligan Significantly Alleviate Liver Damage of Rats Caused by Carbon Tetrachloride in Combination with Kava. J. Food Drug Anal..

[17] Jasemine S., Srivastava R.S., Singh S.K. (2007). Hepatoprotective effect of crude extract and isolated ligan of Justicia simplex Against CCl4-induced hepatotoxicity. Pharm. Biol..

[18] Leary S., Underwood W., Anthony R., Cartner S., Grandin T., Greenacre C., McCrackin M.A., Meyer B., Miller D., Shearer J. (2020). The AVMA Guidelines for the Euthanasia of Animals: 2020 edition.

[19] Thilagavathi R., Begum S.S., Varatharaj S.D., Balasubramaniam A.K., George J.S., Selvam C. (2023). Recent insights into the hepatoprotective potential of medicinal plants and plant-derived compounds. Phytother. Res..

[20] De Silva S.F., Alcorn J. (2019). Flaxseed Ligan as Important Dietary Polyphenols for Cancer Prevention and Treatment: Chemistry, Pharmacokinetics, and Molecular Targets. Pharm. Base..

[21] Senizza A., Rocchetti G., Mosele J.I., Patrone V., Callegari M.L., Morelli L., Lucini L. (2020). Ligan and Gut Microbiota: An Interplay Revealing Potential Health Implications. Molecules.

[22] Carraro J.C.C., Dantas M.I.D., Espeschit A.C.R., Martino H.S.D., Ribeiro S.M.R. (2012). Flaxseed and Human Health: Reviewing Benefits and Adverse Effects. Food Rev. Int..

[23] Prasad K. (2005). Effect of chronic administration of lignan complex isolated from flaxseed on the hemopoietic system. Mol. Cell Biochem..

[24] Billinsky J., Glew R.A., Cornish S.M., Whiting S.J., Thorpe L.U., Alcorn J., Paus-Jenssen L., Hadjistavropoulos T., Chilibeck P.D. (2013). No evidence of hypoglycemia or hypotension in older adults during 6 months of flax lignan supplementation in a randomized controlled trial: A safety evaluation. Pharm. Biol..

[25] Dixon M.F., Fulker M.J., Walker B.E., Kelleher J., Losowsky M.S. (1975). Serum transaminase levels after experimental paracetamol-induced hepatic necrosis. Gut.

[26] Futter L.E., Al-Swayeh O.A., Moore P.K. (2001). A comparison of the effect of nitroparacetamol and paracetamol on liver injury. Brit J. Pharmacol..

[27] Soliman G.A., Yusufoglu H., Tatli-Cankaya I., Abdel-Rahman R.F., Anul S.A., Akaydin G. (2016). Hepatoprotective activities of Lappula barbata and Plantago holosteum against paracetamol induced liver damage in rats and their in vitro antioxidant effects. Planta Med..

[28] Alshawsh M.A., Abdulla M.A., Ismail S., Amin Z.A. (2011). Hepatoprotective Effects of Orthosiphon stamineus Extract on Thioacetamide-Induced Liver Cirrhosis in Rats. Evid.-Based Complement. Altern. Med..

[29] Bellamakondi P.K., Godavarthi A., Ibrahim M. (2018). Caralluma umbellata Haw. Protects liver against paracetamol toxicity and inhibits CYP2E1. Bioimpacts..

[30] Bourdeaux C., Bewley J. (2007). Death from paracetamol overdose despite appropriate treatment with N-acetylcysteine. Emerg. Med. J..

[31] Ahmad M.M., Rezk N.A., Fawzy A., Sabry M. (2019). Protective effects of curcumin and silymarin against paracetamol induced hepatotoxicity in adult male albino rats. Gene.

[32] Landete J.M. (2012). Plant and mammalian lignans: A review of source, intake, metabolism, intestinal bacteria and health. Food Res. Int..

[33] Eeckhaut E., Struijs K., Possemiers S., Vincken J.P., De Keukeleire D., Verstraete W. (2008). Metabolism of the lignan macromolecule into enterolignans in the gastrointestinal lumen as determined in the simulator of the human intestinal microbial ecosystem. J. Agric. Food Chem..

[34] Fuentealba C., Figuerola F., Estévez A.M., Bastíasc J.M., Muñoz O. (2014). Bioaccessibility of lignans from flaxseed (L.) determined by single-batch simulation of the digestive process. J. Sci. Food Agric..

[35] Quartieri A., García-Villalba R., Amaretti A., Raimondi S., Leonardi A., Rossi M., Tomàs-Barberan F. (2016). Detection of novel metabolites of flaxseed lignans in vitro and in vivo. Mol. Nutr. Food Res..

[36] Landete J.M., Arqués J., Medina M., Gaya P., de Las Rivas B., Muñoz R. (2016). Bioactivation of Phytoestrogens: Intestinal Bacteria and Health. Crit. Rev. Food Sci..

[37] Peirotén A., Gaya P., Alvarez I., Bravo D., Landete J.M. (2019). Influence of different lignan compounds on enterolignan production by and strains. Int. J. Food Microbiol..

[38] Kamal-Eldin A., Peerlkamp N., Johnsson P., Andersson R., Andersson R.E., Lundgren L.N., Åman P. (2001). An oligomer from flaxseed composed of secoisolariciresinoldiglucoside and 3-hydroxy-3-methyl glutaric acid residues. Phytochemistry.

[39] Shahidi F., Ambigaipalan P. (2015). Phenolics and polyphenolics in foods, beverages and spices: Antioxidant activity and health effects—A review. J. Funct. Foods.

[40] Yang C., Qiao Z.X., Xu Z.X., Wang X., Deng Q.C., Chen W.C., Huang F.H. (2021). Algal Oil Rich in Docosahexaenoic Acid Alleviates Intestinal Inflammation Induced by Antibiotics Associated with the Modulation of the Gut Microbiome and Metabolome. J. Agric. Food Chem..

[41] Hussain S., Ashafaq M., Alshahrani S., Bokar I.A.M., Siddiqui R., Alam M.I., Taha M.M.E., Almoshari Y., Alqahtani S.S., Ahmed R.A. (2023). Hepatoprotective Effect of Curcumin Nano-Lipid Carrier against Cypermethrin Toxicity by Countering the Oxidative, Inflammatory, and Apoptotic Changes in Wistar Rats. Molecules.

[42] Mar A.N., Adr C., Dana M.M., Mar R.P., Cor L.B., Emi C.B., Anc D.B. (2020). Probiotic Bacillus Spores Protect Against Acetaminophen Induced Acute Liver Injury in Rats. Nutrients.

[43] Cho S.Y., Kim J., Lee J.H., Sim J.H., Cho D.H., Bae I.H., Lee H., Seol M.A., Shin H.M., Kim T.J. (2017). Modulation of gut microbiota and delayed immunosenescence as a result of syringaresinol consumption in middle-aged mice. Sci. Rep..

[44] Taibi A., Ku M., Lin Z., Gargari G., Kubant A., Lepp D., Power K.A., Guglielmetti S., Thompson L.U., Comelli E.M. (2021). Discriminatory and cooperative effects within the mouse gut microbiota in response to flaxseed and its oil and lignan components. J. Nutr. Biochem..

[45] Badger R., Aho K., Serve K. (2021). Short-term exposure to synthetic flaxseed lignan LGM2605 alters gut microbiota in mice. Microbiologyopen.

[46] Corona G., Kreimes A., Barone M., Turroni S., Brigidi P., Keleszade E., Costabile A. (2020). Impact of ligan in oilseed mix on gut microbiome composition and enterolignan production in younger healthy and premenopausal women: An in vitro pilot study. Microb. Cell Fact..

[47] Qin J.Y., Feng Y., Lu X.J., Zong Z.Y. (2021). Precise Species Identification for Acinetobacter: A Genome-Based Study with Description of Two Novel Acinetobacter Species. Msystems.

[48] Nemec A., Dijkshoorn L., Cleenwerck I., De Baere T., Janssens D., van der Reijden T.J.K., Jezek P., Vaneechoutte M. (2003). Acinetobacter parvus sp nov., a small-colony-forming species isolated from human clinical specimens. Int. J. Syst. Evol. Microbiol..

[49] Kiu R., Caim S., Alcon-Giner C., Belteki G., Clarke P., Pickard D., Dougan G., Hall L.J. (2017). Preterm Infant-Associated Clostridium tertium, Clostridium cadaveris, and Clostridium paraputrificum Strains: Genomic and Evolutionary Insights. Genome Biol. Evol..

[50] Walker A., Pfitzner B., Harir M., Schaubeck M., Calasan J., Heinzmann S.S., Turaev D., Rattei T., Endesfelder D., Zu Castell W. (2017). Sulfonolipids as novel metabolite markers of Alistipes and Odoribacter affected by high-fat diets. Sci. Rep..

[51] Zhang T., Li Q.Q., Cheng L., Buch H., Zhang F. (2019). Akkermansia muciniphila is a promising probiotic. Microb. Biotechnol..

[52] Han K.H., Hashimoto N., Fukushima M. (2016). Relationships among alcoholic liver disease, antioxidants, and antioxidant enzymes. World J. Gastroentero..

[53] Bayram H.M., Majoo F.M., Ozturkcan A. (2021). Polyphenols in the prevention and treatment of non-alcoholic fatty liver disease: An update of preclinical and clinical studies. Clin. Nutr. Espen..

[54] Yang Y.P., Jian Y.Q., Cheng S.W., Jia Y.Z., Liu Y.B., Yu H.H., Cao L., Li B., Peng C.Y., Choudhary M.I. (2021). Dibenzocyclooctadiene ligan from Kadsura coccinea alleviate APAP-induced hepatotoxicity via oxidative stress inhibition and activating the Nrf2 pathway in vitro. Bioorg. Chem..

[55] Kang D., Shao Y.H., Zhu Z.P., Yin X.X., Shen B.Y., Chen C., Xu Y.F., Shen J.J., Li H.F., Li X.N. (2019). Systematically identifying the hepatoprotective ingredients of schisandra lignan extract from pharmacokinetic and pharmacodynamic perspectives. Phytomedicine.

[56] Paulusma C.C., Lamers W.H., Broer S., van de Graaf S.F.J. (2022). Amino acid metabolism, transport and signalling in the liver revisited. Biochem. Pharmacol..

[57] de la Puerta C., Arrieta F.J., Balsa J.A., Botella-Carretero J.I., Zamarron I., Vazquez C. (2010). Taurine and glucose metabolism: A review. Nutr. Hosp..

[58] Kunst C., Schmid S., Michalski M., Tumen D., Buttenschon J., Muller M., Gulow K. (2023). The Influence of Gut Microbiota on Oxidative Stress and the Immune System. Biomedicines.

[59] Hanafy M.M., Lindeque J.Z., El-Maraghy S.A., Abdel-Hamid A.H.Z., Shahin N.N. (2021). Time-based investigation of urinary metabolic markers for Type 2 diabetes: Metabolomics approach for diabetes management. Biofactors.

[60] Glatz J.F.C., Luiken J.J.F.P., Bonen A. (2010). Membrane Fatty Acid Transporters as Regulators of Lipid Metabolism: Implications for Metabolic Disease. Physiol. Rev..

[61] Dean B., Chang S., Doss G.A., King C., Thomas P.E. (2004). Glucuronidation, oxidative metabolism, and bioactivation of enterolactone in rhesus monkeys. Arch. Biochem. Biophys..

[62] Drygalski K., Berk K., Charytoniuk T., Ilowska N., Lukaszuk B., Chabowski A., Konstantynowicz-Nowicka K. (2017). Does the enterolactone (ENL) affect fatty acid transporters and lipid metabolism in liver?. Nutr. Metab..

[63] Puri P., Mirshahi F., Siddiqui M.S., Boyett S., Sargeant C.C., Joyce A., Luketic V.A., Sanyal A.J. (2015). Decreased Fecal Microbial Metabolite Enterolactone is a Novel Inflammatory and Oxidative Stress Related Biomarker of Alcoholic Liver Disease. Gastroenterology.

