# Supplementary material for: Flaxseed Lignan Alleviates the Paracetamol-Induced Hepatotoxicity Associated with Regulation of Gut Microbiota and Serum Metabolome
Source: Nutrients. 2024 Jan 18;16(2):295. doi: 10.3390/nu16020295 (PMC10821007; doi:10.3390/nu16020295)

Supplemental file

Table S1. The composition of a standard diet.

AIN93M	gm%	kcal%
Fat	4	9.4
Protein	14.2	14.7
Carbohydrate	73.1	75.9
kcal/gm		3.85
Ingredient	gm	kcal
Casein, 30 Mesh	140	560
L-Cystine	1.8	7.2
Corn Starch	495.692	1983
Maltodextrin 10	125	500
Sucrose	100	400
Cellulose	50	0
Soybean Oil	40	360
t-Butylhydroquinone	0.008	0
Mineral Mix S10022M	35	0
Vitamin Mix V10037	10	40
Choline Bitartrate	2.5	0
Total	1000	3850
*Soluble fiber/carbohydrate	540.85	
*Insoluble fiber/carbohydrate	50.49	

Figure S1 Super class distribution of significantly changed metabolites in each pair-group.

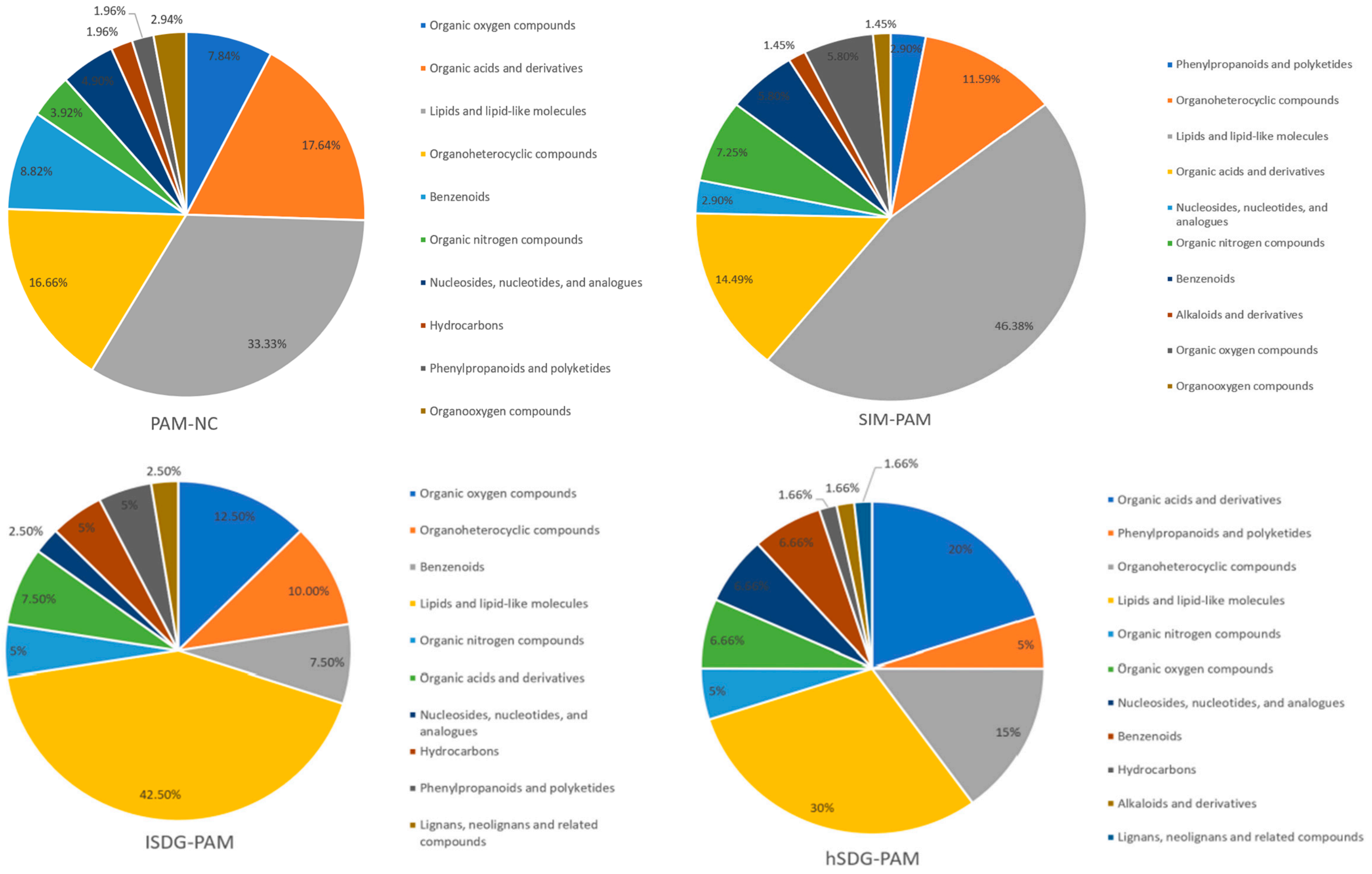

Figure S2 Heatmap of Spearman's correlation between changed metabolites and biochemical parameters.

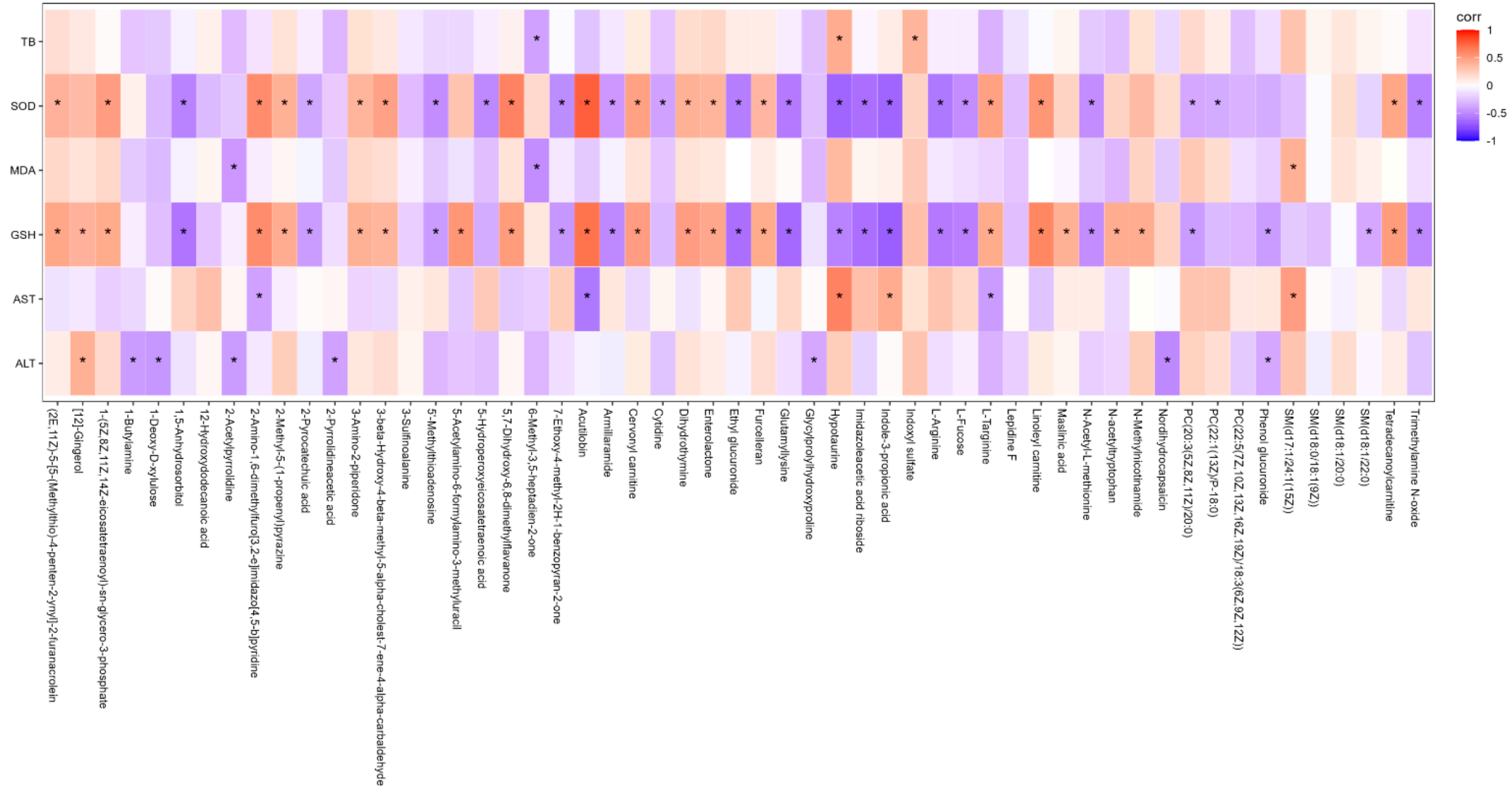

Supplement: Supplementary file 1 [file nutrients-16-00295-s001.zip › nutrients-2733253-supplementary.pdf]
